# When depression envelops frontotemporal dementia: The differential diagnostic frame through a case report

**DOI:** 10.1192/j.eurpsy.2022.1664

**Published:** 2022-09-01

**Authors:** U. Altunoz, J. Menstell, M. Ziegenbein

**Affiliations:** 1 Wahrendorff Clinic, Centre Of Transcultural Psychiatry & Psychotherapy, Sehnde, Germany; 2 Hannover Medical School, Research Group For Social And Transcultural Psychiatry & Psychotherapy, Hannover, Germany; 3 Wahrendorff Clinic, Department Of Psychiatry & Psychotherapy, Sehnde, Germany

**Keywords:** differential diagnosis, Depression, frontotemporal dementia

## Abstract

**Introduction:**

Frontotemporal dementia (FTD) is common in presenile population. The overlapping symptoms with other psychiatric disorders can lead to wrong/late diagnosis which cause delays/difficulties regarding case-management. Especially, long-standing and/or late-onset depression can descriptively envelop bvFTD (behavioral-variant) and leads to unnecessary treatments and increased distress. It’s important to implement a descriptive diagnostic algorithm which will help clinicians to distinguish the phenomenology of these disorders.

**Objectives:**

This presentation aims to call attention of the clinicians/researchers to an elaborated effort concerning differential diagnosis of two common disorders with overlapping features through a case-study of a 59-year-old male patient.

**Methods:**

One case from an inpatient unit of a psychiatric clinic in Lower Saxony, Germany will be reported.

**Results:**

Case: The patient was referred to our acute-psychiatric-ward from the day-clinic-unit because of treatment-resistant, severe and long-lasting depressive symptoms. He was depressed, desperate, hopeless, listless and had suicidal thoughts. During the first days of treatment, symptoms like apathy, bad hygiene, weird eating-behavior, urinary incontinence, lack of empathy, language disorders and other behavioral symptoms were evident. Brain-MRI yielded frontotemporal lobar atrophy. Trail-Making-Test and Frontal-Assessment-Battery showed pronounced impairment of executive functions. Mini mental state examination and DemTect yielded light to moderate memory dysfunction. Diagnostic Criteria for Probable bvFTD (International-Consensus-Criteria) were fulfilled.

**Conclusions:**

The diagnosis of bvFTD enabled a rapid assignment of a legal representative and relieved the long-lasting discomfort of the patient and his family that was caused by multiple unsuccessful treatment trials against depression. The differential diagnostic frame between bvFTD and depression will be discussed in view of the current literature.

**Disclosure:**

No significant relationships.

